# Country Quarantine During COVID-19: Critical or Not?

**DOI:** 10.1017/dmp.2020.384

**Published:** 2020-10-13

**Authors:** Noosha Samieefar, Reza Yari Boroujeni, Mahnaz Jamee, Melika Lotfi, Mohammad Rasul Golabchi, Alireza Afshar, Hamidreza Miri, Mohammad Amin Khazeei Tabari, Pouya Darzi, Morteza Abdullatif Khafaie, Bagher Amirheidari, Amin Tamadon, Niloofar Rambod Rad, Nastaran Samimi, Mojtaba Farjam, Fatemeh Shiravi, Narges Farshidi, Mojtaba Hedayati Ch, Donya Doostkamel, Radin Alikhani, Mahboobeh Razmkhah, Saeed Abdollahifard, Rasoul Nasiri Kalmarzi, Roya Kelishadi, Hosseinali Khazaei, Asghar Aghamohammadi, Farzaneh S. Jafari Mousavi, Morteza Shamsizadeh, Arash Khojasteh, Nima Rezaei

**Affiliations:** Student Research Committee, School of Medicine, Shahid Beheshti University of Medical Sciences, Tehran, Iran; USERN Office, School of Advanced Technologies in Medicine, Shahid Beheshti University of Medical Sciences, Tehran, Iran; USERN Office, Alborz University of Medical Sciences, Karaj, Iran; USERN Office, Zanjan University of Medical Sciences, Zanjan, Iran; USERN Office, Isfahan University of Medical Sciences, Isfahan, Iran; USERN Office, The Persian Gulf Biomedical Sciences Research Institute, Bushehr, Iran; USERN Office, Hormozgan University of Medical Sciences, Bandar Abbas, Iran; USERN Office, Mazandaran University of Medical Sciences, Sari, Iran; Social Determinants of Health Research Center, Ahvaz Jundishapur University of Medical Sciences, Ahvaz, Iran; USERN Office, Ahvaz Jundishapur University of Medical Sciences, Ahvaz, Iran; Department of Pharmaceutical Biotechnology, Faculty of Pharmacy, Kerman University of Medical Sciences, Kerman, Iran; USERN Office, Kerman University of Medical Sciences, Kerman, Iran; The Persian Gulf Marine Biotechnology Research Center, The Persian Gulf Biomedical Sciences Research Institute, Bushehr University of Medical Sciences, Bushehr, Iran; USERN Office, Islamic Azad University Medicine Faculty, Mashhad, Iran; USERN Office, Noncommunicable Diseases Research Center, Fasa University of Medical Sciences, Fasa, Iran; Noncommunicable Diseases Research Center, Fasa University of Medical Sciences, Fasa, Iran; Department of Microbiology, Faculty of Medicine, Guilan, University of Medical Sciences, Rasht, Guilan, Iran; USERN Office, Guilan University of Medical Sciences, Guilan, Iran; USERN Office, Ardabil University of Medical Sciences, Ardabil, Iran; USERN Office, Shiraz University of Medical Sciences, Shiraz, Iran; USERN Office, Kurdistan University of Medical Sciences, Kurdistan, Iran; Child Growth and Development Research Center, Research Institute for Primordial Prevention of Non-Communicable Disease, Isfahan University of Medical Sciences, Isfahan, Iran; USERN Office, Research Institute for Primordial Prevention of Non-Communicable Disease, Isfahan University of Medical Sciences, Isfahan, Iran; Clinical Immunology Research Center of Zahedan University of Medical Sciences, Zahedan, Iran; USERN Office, Zahedan University of Medical Sciences, Zahedan, Iran; Research Center for Immunodeficiencies, Children’s Medical Center, Tehran University of Medical Sciences, Tehran, Iran; USERN RCI Lab, Research Center for Immunodeficiencies, Tehran University of Medical Sciences, Tehran, Iran; Department of Medical Surgical Nursing, School of Nursing and Midwifery, Hamedan University of Medical Sciences, Hamedan, Iran; USERN Office, Hamadan University of Medical Sciences, Hamedan, Iran; Department of Oral and Maxillofacial Surgery, Dental Research Center, Research Institute of Dental Sciences, School of Dentistry, Shahid Beheshti University of Medical Sciences, Tehran, Iran; USERN Headquarters, Universal Scientific Education and Research Network (USERN), Tehran, Iran

**Keywords:** COVID-19, Iran, patient isolation, quarantine, SARS-CoV-2

The new coronavirus, or severe acute respiratory syndrome coronavirus 2 (SARS-CoV-2), which causes coronavirus disease (COVID-19), emerging from Wuhan, China, is the subject of attention in these days and the world news headlines.^1^ The first case was reported on December 31, 2019, and the disease was declared a Public Health Emergency of International Concern by the World Health Organization (WHO), a month later, on January 30, 2020.^2^

Iran is one of the most affected countries with more than 290 000 confirmed cases of COVID-19 (in April 29) and unfortunately more than 15 000 associated deaths. According to the geographic distribution data, all 31 provinces in Iran have been affected. The first 2 cases were announced in Qom and then north-central provinces became the hotspot regions, mainly capital cities.

There is no specific medication or vaccine available for this infection, and other COVID-19 outbreaks seem to be inevitable; this emphasizes the need for finding the most beneficial preventive measures.^1^ Isolation, quarantine, social distancing, and community containment are now the available options. Isolation for the purpose of symptomatic and non-infected individuals’ segregation does not appear to be sufficient alone due to the long incubation period of COVID-19. Quarantine as a previous successful measure during the SARS epidemic control in 2003, aiming to restrict the movement of suspected persons (maybe not infected or infected but without symptoms), could be beneficial. Social distancing is another option in which gatherings are reduced in order to avoid close contact of non-detected cases and with the community. The last strategy is community containment, as chosen by the Government of China. It is the restriction of the whole society and limiting the traffic to vital needs only.^3^ However, a mass quarantine may increase anxiety, especially among those having previous psychiatric problems and the elderly, even causing other health problems.^4^

In Iran, there is community transmission, which means the infection is expanding in numerous independent cluster.^5^ Therefore, social distancing as done with the closure of schools and universities is beneficial and was fulfilled on February 23. Education guidelines, traveler screenings, charitable donations, the self-assessment system, and travel control may have also contributed to this outcome. The result was the decreasing number of confirmed cases in April. From the beginning of the epidemic, the government has emphasized social distancing rather than mass quarantine. Traveling between cities, although in a decline compared with that during previous years, was still taking place. In April 2020, the government decided to move the policy to smart social distancing while resuming social activities as before.^6^ However, due to the reopening of the offices and increased hubbub, the number of infected individuals has been increasing with a stable trend.

Some Asian countries have implemented successful strategies of pandemic control. The strategies were based on mostly transmission control via isolation and lockdowns, like what the Chinese Government did in Wuhan.^7^ Although quarantining faces numerous obstacles, evidence supports its efficacy with emerging infectious diseases.^8^ In Iran, the increased rate of transmission after returning the society to normal social activities resulted in an incremental trend. In conclusion, the best option available right now is transmission control. Infection cases must be detected promptly, isolated, and treated. However, isolation alone is not the answer; quarantine seems to be an advantageous tool, but its implementation needs resources. Furthermore, the entire society must be responsible and also educated about the disease. They should be aware of the alarming signs of the infection and should voluntarily quarantine themselves when having dubious mild symptoms or if they become exposed to a person who is infected with the new coronavirus.

A group collaboration and awareness are needed to fight the pandemic successfully. “May the disease be controlled by all working side by side, as human beings are members of one another.”
